# AFTG-Net: A Deep Attention-based Fusion Framework of Topological and Gradient Features for Pathological Image Analysis

**DOI:** 10.21203/rs.3.rs-6710077/v1

**Published:** 2025-07-11

**Authors:** Taymaz Akan, Fatih Gelir, Richa Aishwarya, Md. Shenuarin Bhuiyan, Mohammad Alfrad Nobel Bhuiyan

**Affiliations:** 1 Department of Medicine, LSU Health Shreveport, Shreveport, LA, USA; 2 Department of Software Engineering, Faculty of Engineering, Topkapı University, Istanbul, Turkey; 3 Department of Pathology and Translational Pathobiology, LSU Health Shreveport, Shreveport, LA, USA

**Keywords:** Muscle Disease Diagnosis, Histogram of Oriented Gradients, Topological data analysis, Feature Fusion

## Abstract

Skeletal muscle pathology is observed by structural disruptions in sarcomeres, increased central nuclei, and changes in myofiber cross-sectional area. In order to classify amyotrophic lateral sclerosis (ALS), diabetes, and healthy controls, pathologists examine the changes in myofiber size using Wheat Germ Agglutinin (WGA) stained histopathological images of various skeletal muscles (quadriceps, gastrocnemius, tibialis anterior, extensor digitorum longus, and soleus). Histological image analysis of skeletal muscle pathology is laborious and subject to inter- and intra-user variability, which can affect diagnosis accuracy and consistency. Conventional techniques like ImageJ-based tools are time-consuming and produce varying outcomes due to their manual cell counting, segmentation, and thresholding. This study introduces AFTG-Net, an attention-based machine learning framework that classifies skeletal muscle histopathological images using complementary geometric and topological descriptors. The model uses globally structural information from Topological Data Analysis (TDA) based on persistent homology and local edge and texture patterns from the Histogram of Oriented Gradients. We suggest a cross-weighted fusion approach that uses cosine similarity to adaptively balance the contributions of these heterogeneous features in order to improve their discriminative power. This integration enables the model to effectively distinguish pathological changes associated with amyotrophic lateral sclerosis (ALS) and Type I diabetes from healthy muscle tissue. We conducted comprehensive comparisons with various state-of-the-art and baseline methods, such as traditional feature-based and deep learning models. We assessed all models by analyzing WGA-stained skeletal muscle images from wild-type and disease models (G93A*SOD1 for ALS and Akita for type 1 diabetes). AFTG-Net outperformed all other models by achieving 92% classification accuracy in distinguishing healthy and diseased muscle fibers. By reducing human intervention, subjectivity, and analysis time, AFTG-Net improves scalability and diagnostic consistency, making it a valuable tool for both biomedical research and clinical practice.

## 1 Introduction

Histopathological image analysis remains the gold standard for diagnosing skeletal muscle disorders, enabling detailed assessment of structural and cellular abnormalities indicative of disease. WGA-stained muscle biopsy images, which highlight extracellular matrix components, contain rich histopathological information and are commonly used in clinical evaluation. Histopathological image classification, particularly of WGA-stained skeletal muscle tissues affected by amyotrophic lateral sclerosis (ALS) and Type I diabetes, can significantly assist pathologists by enabling more accurate, efficient, and objective diagnoses of disease-related structural abnormalities.

Currently, an estimated 1.7 billion individuals worldwide are affected by musculoskeletal disorders, and the prevalence is on the rise as a result of aging populations and changing lifestyles [1]. These conditions encompass a wide variety of diseases that affect the connective tissues surrounding skeletal muscles, bones, and joints. Significant locomotor impairment can result from any of these components’ malfunction, which can impact both fine motor control and mobility [1].

Among the integral parts of the musculoskeletal system, skeletal muscle plays a particularly vital role. Composed of tightly packed muscle fibers segmented into organized contractile myofibrils, skeletal muscle exhibits a complex and highly structured architecture [2]. Myopathies, neuromuscular diseases, sarcopenia, chronic traumatic limb injuries (CTLI), and muscular dystrophies are among the skeletal muscle disorders associated with disruptions in this structure, whether caused by changes in muscle fibers or genetic mutations affecting contractile proteins [3–6].

The pathological progression of muscle diseases is typically characterized by observable histological changes. These include the infiltration of immune cells, sarcomere disorganization, fibrosis, vascular compromise, and distortions in fiber shape, as well as variations in myofiber diameter (atrophy or hypertrophy), central nucleation that indicates muscle repair or degeneration [6–11]. Pathologists utilize these morphological signatures as critical diagnostic indicators to differentiate between healthy and diseased muscle tissues [12].

Muscle biopsies slide microscopic analysis is still a vital diagnostic tool, offering important information about tissue structure and cellular anomalies linked to inflammatory and degenerative diseases [12][13]. Manual evaluation is labor-intensive, subjective, and susceptible to intra- and inter-observer variability, despite its diagnostic value [8, 14]. Moreover, the rising number of biopsy cases and the global shortage of qualified pathologists are putting healthcare systems under more pressure. These challenges have accelerated interest in computational solutions, particularly digital pathology, which offers promising avenues for automated tissue analysis.

Digital pathology can reduce diagnostic errors, enhance reproducibility, and alleviate the burden on clinical experts by digitizing histological slides and employing sophisticated image processing techniques. In the context of musculoskeletal disease diagnosis, this approach is particularly pertinent, as it is imperative to accurately evaluate subtle histopathological changes in order to provide timely and effective intervention [15, 16].

In recent years, machine learning (ML) techniques have emerged as effective tools in the field of digital pathology [17–21]. The automatic learning of discriminative features from histopathological images has been a remarkable success for ML models, particularly those based on computer vision. This has enabled the reliable detection of anomalies, segmentation, and classification functions. The consistency and speed of manual evaluation are frequently surpassed by the ability of these models to identify intricate morphological patterns that are associated with myopathic changes via training.

Topological Data Analysis (TDA) is a mathematical approach that is employed to explore the shape and structure of data by analyzing its topological features, including connectivity and holes, across various scales [22]. In the past decade, TDA has achieved significant success in medical image analysis, improving the accuracy of diagnosis and extracting significant features from complex data [23–29]. TDA proficiently reveals hidden topological patterns in images and converts them into significant characteristics, offering knowledge about the fundamental structure of complex data. Employing a technique from TDA known as persistent homology (PH), we could transform these patterns into significant image characteristics that classifier models can utilize for highly precise classification [30, 31]. TDA has shown promise in a variety of medical research areas, including oncology, ophthalmology, dermatology, and pathology, providing structural and morphological insights [32–38], but its potential for broader medical picture analysis remains untapped.

Alongside TDA, the Histogram of Oriented Gradients (HOG) has emerged as a prevalent feature descriptor in image analysis, thanks to its capacity to capture local edge structures and spatial gradients [39]. By encoding the distribution of gradient orientations within localized regions of an image, HOG effectively represents contours, shapes, and textures. It is particularly suitable for medical image analysis tasks that require diagnostically relevant fine structural detail due to its low computational complexity and resistance to changes in lighting or geometry. Furthermore, HOG has been successfully employed to identify anatomical features, lesions, or textural irregularities in a variety of biomedical domains, including mammography, brain imaging, and retinal analysis [40–45].

While topological features provide a global view of image structure, they may fail to recognize subtle local variations, such as subtle fiber shape irregularities, endomysial disruptions, or microstructural lesions, which are crucial in histopathological images. On the other hand, the HOG is a feature descriptor that is intended to capture local shape and texture information by analyzing the distribution of gradient orientations within localized regions of an image. HOG is particularly useful for illustrating the fine-grained patterns that are frequently observed in histopathological images, as it is particularly effective at representing structural edges and contours. Despite the fact that each technique has unique benefits, using either alone may not fully capture the intricacy of skeletal muscle pathology. This motivates the development of a feature fusion strategy that can combine the local structural sensitivity of HOG with the global structural awareness of TDA to provide a more discriminative and comprehensive feature representation.

To the best of our knowledge, no prior studies have explored a principled fusion of these two complementary descriptors for image analysis. In this study, we propose a cross-weighted fusion strategy that adaptively integrates TDA and HOG features. The method computes the cosine similarity between the normalized feature vectors and uses this similarity to dynamically determine their contribution to a final fused vector. This approach enables the model to achieve a unified representation for downstream classification by balancing the global topological patterns and local geometric structures based on their alignment in a given image. We have shown that the classification accuracy and consistency of our skeletal muscle pathology datasets are enhanced when this approach is implemented in comparison to the use of either descriptor independently.

## 2 Materials and methods

### 2.1 Animal Models and Tissue Preparation

Skeletal muscle samples were obtained from two established mouse models of disease: G93A*SOD1 transgenic mice, a model of amyotrophic lateral sclerosis (ALS), and C57BL/6-Ins2Akita/J (Akita) mice, a model for Type I diabetes. Wild-type and G93A*SOD1 non-carrier mice on a C57BL/6 background were used as healthy controls. Both the disease models and their respective control mice were purchased from The Jackson Laboratories (Bar Harbour, ME, USA). All mice were maintained in standard housing conditions, which included temperature-controlled environments, 12-hour light-dark cycles, unlimited access to water, and a typical chow diet. Animal care and experimental procedures were conducted in compliance with the NIH Guide for the Care and Use of Laboratory Animals, as approved by the LSU Health-Shreveport Institutional Animal Care and Use Committee. The specifics of muscle dissection and sample processing have been previously detailed [46].

### 2.2 WGA Staining and Microscopy

Skeletal muscle tissues were collected from five distinct anatomical regions: quadriceps, gastrocnemius, soleus, extensor digitorum longus (EDL), and tibialis anterior (TA). Following isoflurane anesthesia, tissues were extracted bilaterally, fixed in formalin, and paraffin-embedded to prepare histological blocks. Thin sections (5 μm) were cut and subjected to deparaffinization and rehydration. Antigen retrieval was performed by boiling in 10 mmol/L sodium citrate buffer (pH 6.0) at 100°C. After blocking in a solution containing 1% bovine serum albumin, 0.1% cold water fish skin gelatin, and 1% Tween-20 in PBS, sections were incubated with Alexa Fluor 488-conjugated wheat germ agglutinin (WGA, 5 μg/mL) for 1 hour to label cell membranes. The nuclei were counterstained with DAPI for a duration of 5 minutes. The slides were washed in PBS and mounted using Vectashield mounting medium. The Nikon A1R confocal microscope (20× objective) was employed to conduct imaging, and Nikon NIS Elements (v4.13.04) was employed to analyze the images. Previous research has provided a comprehensive description of the complete staining and imaging protocol [47, 48].

### 2.3 Dataset and Classification Framework

The final dataset included five different muscle types per subject, including WGA-stained muscle sections from Akita, G93A*SOD1, and wild-type mice. Centrally located nuclei, an increase in small or irregularly shaped fibers, and altered myofiber morphology are among the characteristic pathological alterations seen in G93A*SOD1 mice, a model that is representative of ALS [7] [11] [49]. On the other hand, healthy muscle fibers are characterized by polygonal geometry, peripheral nuclei, and variable cross-sectional area (CSA) [11]. The Akita mouse, which serves as a model for Type I diabetes, is distinguished by histological abnormalities and muscle dysfunction [50, 51]. All images were labeled as either disease (ALS or diabetic muscle) or healthy and used for model development and evaluation. The dataset was divided into training and test sets for performance benchmarking of the classification framework.

## 3 Automated Image Analysis

### 3.1 Topological Data Analysis

In this paper, we have applied PH as an effective feature extraction method for images. PH is a fundamental method in TDA, allowing us to analyze the hidden patterns in the data by adjusting a scale parameter [52]. We used cubical persistence [53], a form of PH designed for image data. It analyzes topological features like connected components, holes, and cavities in images by working with cubical complexes, which naturally fit the grid structure of image pixels or voxels.

PH can be broken down into three main steps. The first step is the filtration. It helps to build a bridge between data and its topological features. It also provides information on the structure of the data. Later, to capture topological features in a dataset, PH is converted to persistence diagrams (PD). These diagrams distinguish between stable and significant features by plotting each topological feature as a point with its birth (when the feature appears) and death (when the feature disappears) on the axes. The distance of points from the diagonal in a PD, where birth equals death, quantifies the features’ persistence and stability. Vectorization is the last step that transforms these data from PDs into vector formats usable with conventional ML algorithms [54, 55].

**Figure 1.**
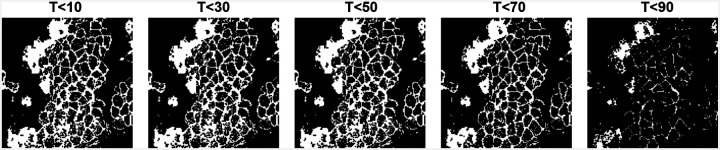
Binary images $I_{10},\;I_{30},\;I_{50},\;I_{70}$, and $I_{90}$ were generated from a dataset image by applying threshold values of 10, 30, 50, 70, and 90.

In image analysis, filtrations are constructed differently due to the grid-based structure of image data. A filtration is generated by creating a sequence of nested binary images (cubical complexes), based on intensity thresholds applied to a selected color channel or grayscale version of the image. For a given image I with pixel intensities $\beta_{ij}\in [0,255]$, a set of thresholds $\left\{p_{t}\right\}$ is chosen (typically between 50 and 100 values) to span the intensity range. In sublevel filtration, pixels are included in the binary image if their intensity is less than or equal to a given threshold, forming a nested sequence $I_{1}\subset I_{2}\subset \ldots \subset I_{l}$ (as illustrated in Figure 1). Alternatively, in superlevel filtration, the process is reversed: pixels are included if their intensities are greater than or equal to descending thresholds. These filtrations provide a framework to analyze topological changes in image structure across scales [56].

In persistent homology (PH), threshold selection is essential because it dictates the degree of detail in the topological features that are extracted. Too few thresholds can cause significant structures to be missed, while utilizing all 255 grayscale levels may produce excessively fine filtrations and trivial outputs. In our study, we selected N=100, which balanced accuracy and efficiency, as higher values did not improve performance.

PDs are used to identify topological features such as connected components (0D) and loops (1D) by tracking their birth and death across grayscale filtrations. As pixel intensities increase, a filtration process records the emergence and merging of features. Each feature is represented as a point in the PD, with birth and death values indicating when it appears and disappears in the sequence. Formally, for dimension k, the diagram is defined calculated per: $PD_{k}(I)=\left\{\left(\alpha_{\text{birth}}(\sigma ),\alpha_{\text{death}}(\sigma )\right)|\;\text{changes}\;\text{at}\;\sigma \right\}$.

**Figure 2.**
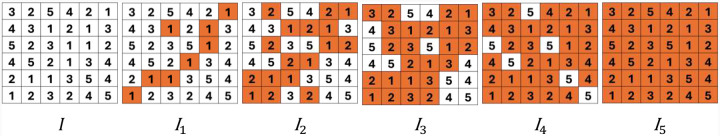
The sublevel filtration is illustrated using a 6 × 6 image with assigned pixel values. This process generates a series of binary images, where each successive image is a subset of the next, forming a nested structure $I_{1}\subset I_{2}\subset \ldots \subset I_{5}$. Each image in the sequence represents a sublevel set, progressively incorporating pixels based on their intensity values.

Figure 2 demonstrates how sublevel filtration reveals topological features in a 6 × 6 image. At the first filtration step $I_{1}$, two connected components (0-dimensional features) emerge. As the threshold increases to $I_{2}$, components merge, reducing their count to one, yielding the 0-D persistence diagram: $PD_{0}(I)=\{(1,\infty ),(1,2\}$. For 1-D features (loops), none appear in $I_{1}$, but one hole is formed at $I_{2}$ which dies at $I_{5}$. Then, two holes are formed at $I_{4}$ both die at $I_{5}$, resulting in: $PD_{1}(I)=\{(2,5),(4,5),(4,5)\}$. Since persistence diagrams consist of unordered pairs and are not directly suitable for machine learning models, they are typically vectorized. One common method is through Betti curves. The Betti function $\beta_{k}\left(p_{t}\right)$ counts the number of active topological features of dimension k at threshold $p_{t}$. This results in a vector ${\vec{\beta }}_{k}=\left[\beta_{k}\left(p_{1}\right),\beta_{k}\left(p_{2}\right),\ldots ,\beta_{k}\left(p_{L}\right)\right]$, where L is the number of thresholds. For example, in Figure 2, the Betti curve for 0D features is ${\vec{\beta }}_{0}(I)=[2,1,1,1,1]$, and for 1D features it is ${\vec{\beta }}_{1}(I)=[0,1,1,3,0]$. These curves summarize how the number of components and holes evolve during the filtration.

### 3.2 Histogram of Oriented Gradients

Histogram of Oriented Gradients (HOG) is a feature descriptor designed to encode local object appearance and shape by capturing the distribution of gradient orientations within localized regions of an image. The method proceeds in three main steps: gradient computation, orientation histogramming, and block-wise normalization.

#### Gradient Computation:

Let I(x,y) denote the intensity at pixel (x,y) in the grayscale image. The gradient vector ∇I(x,y) is computed using first-order derivative filters, typically the Sobel operator: $G_{x}(x,y)=I\left(x+1,y\right)-I(x-1,y),G_{y}(x,y)=I(x,y+1)-I(x,y-1)$. From these components, we compute the gradient magnitude and orientation as: $M(x,y)=\sqrt{G_{x}(x,y{)}^{2}+G_{y}(x,y{)}^{2}},\theta (x,y)={\text{tan}}^{-1}\left(\frac{G_{y}(x,y)}{G_{x}(x,y)}\right)$.

#### Histogram Construction:

Each image is divided into small, non-overlapping cells (e.g., 8 × 8 pixels). For every pixel in a cell, the orientation θ(x,y) is quantized into one of B bins (typically B=9, covering 0° – 180°). The corresponding magnitude M(x,y) is then accumulated into the appropriate orientation bin as $H_{b}=\sum_{(x,y)\in \text{cell}}\;w_{b}(x,y)\cdot M(x,y)$, where $w_{b}(x,y)$ is a weight indicating how much the pixel contributes to bin b, often computed via linear interpolation between neighboring bins.

#### Block Normalization:

To reduce the impact of illumination and contrast variations, local contrast normalization is applied. Cells are grouped into overlapping blocks (e.g., 2 × 2 cells), and each block’s concatenated histogram vector v is normalized per: $v_{\text{norm}}=\frac{v}{\sqrt{\|v{\|}_{2}^{2}+\varepsilon^{2}}}$, where ε is a small constant (e.g., ε=1e-6) to prevent division by zero.

#### Application in WGA-stained muscle images:

**Figure 3.**
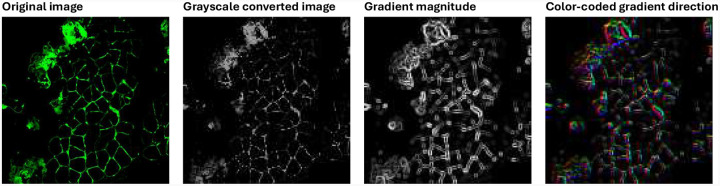
From left to right: (1) Original input image (fluorescence microscopy); (2) Grayscale converted image; (3) Gradient magnitude showing edge strength; (4) Color-coded gradient direction image, where hue represents orientation angles derived from Sobel filters.

In WGA-stained muscle images, HOG is particularly useful for detecting curved edges such as blood vessels and the optic disc boundary. In this study, we extracted HOG features using cell size of 8 × 8 pixels, block size of 2 × 2 cells, stride of 8 pixels, and 9 orientation bins. This configuration provides a dense representation that captures local anatomical structure while maintaining robustness to lighting variations. As shown in Figure 3, the gradient magnitude and color-coded orientation map derived from Sobel filters highlight prominent structural boundaries in the fluorescence microscopy image.

The Histogram of Oriented Gradients (HOG) descriptor was computed using a configuration optimized for 64 × 64 pixel input images. Each image was divided into cells of size 8 × 8 pixels, and blocks were defined as groups of 2 × 2 adjacent cells, covering 16 × 16 pixels. The block stride was set to 8 pixels, allowing blocks to overlap. With this configuration, the image contains 64/8 = 8 cells per row and column. Since each block spans 2 cells and moves in steps of 1 cell, the number of blocks per row and column is 8 − 2 + 1 = 7, resulting in 7 × 7 = 49 blocks in total. Each block includes four cells, and with 9 orientation bins per cell, yields 4 × 9 = 36 features. Therefore, the final HOG feature vector has a dimensionality of 49 × 36 = 1,764 features.

### 3.3 Cross-Weighted Fusion Strategy

We suggest a cross-weighted fusion strategy that adaptively combines two distinct feature representations based on their mutual similarity in order to capitalize on their complementary strengths. This method guarantees that the final descriptor is proportionally contributed to by both representations, while simultaneously preserving robustness in the face of scale and magnitude variations. Normalization, similarity measurement, and a weighted combination based on alignment strength comprise the fusion process.

Let:

- $f_{HOG}\in R^{n}$ be the normalized HOG feature vector
- $f_{\text{TDA}}\in R^{m}$ be the normalized TDA feature vector
- α∈[0,1] be the cosine similarity between the two vectors (on a shared length basis)

Step 1: Normalize vectors
(1) $${\tilde{f}}_{\text{HOG}}=\frac{f_{\text{HOG}}}{\left\|f_{\text{HOG}}\right\|},{\tilde{f}}_{\text{TDA}}=\frac{f_{\text{TDA}}}{\left\|f_{\text{TDA}}\right\|}$$
Step 2: Compute similarity
(2) $$\alpha =\text{cos}(\theta )=\frac{{\tilde{f}}_{\text{HOG}}\cdot {\tilde{f}}_{\text{TDA}}}{\left\|{\tilde{f}}_{\text{HOG}}\right\|\left\|{\tilde{f}}_{\text{TDA}}\right\|}\in [0,1]$$
  Note: If the vectors have different dimensions, truncate both to their minimum length before computing α.
Step 3: Cross-weighted fusion
(3) $$f_{\text{fused}}=\left[\alpha \cdot f_{\text{HOG}}\|(1-\alpha )\cdot f_{\text{TDA}}\right],$$
  where [⋅‖⋅] denotes vector concatenation. The fused feature vector $f_{\text{fused}}\in R^{n+m}$ encodes both local geometric detail and global topological patterns in a way that adapts to their similarity.

### 3.4 Classification

The cross-weighted integration of HOG and TDA produced the fused feature vectors, which yielded 2,564 features overall per sample (1,764 from HOG and 800 from TDA). The 200-dimensional representations that were taken from each RGB channel and grayscale (i.e., 200 × 4 = 800) made up the TDA features. Each sample was then truncated to 2,560 features in order to guarantee dimensional consistency across all input vectors. For the classification task, all samples were numerically encoded in binary form and labeled as either disease or non-disease.

Prior to classification, the dataset was partitioned using stratified 20-fold cross-validation, which preserved the class balance in each training and validation split. This approach ensured that all samples contributed to both model training and evaluation, while preventing data leakage and maintaining robust performance estimation. All processing steps were applied consistently across folds with fixed random seed initialization to enable reproducibility.

#### 3.4.1 Model Architecture

**Figure 4.**
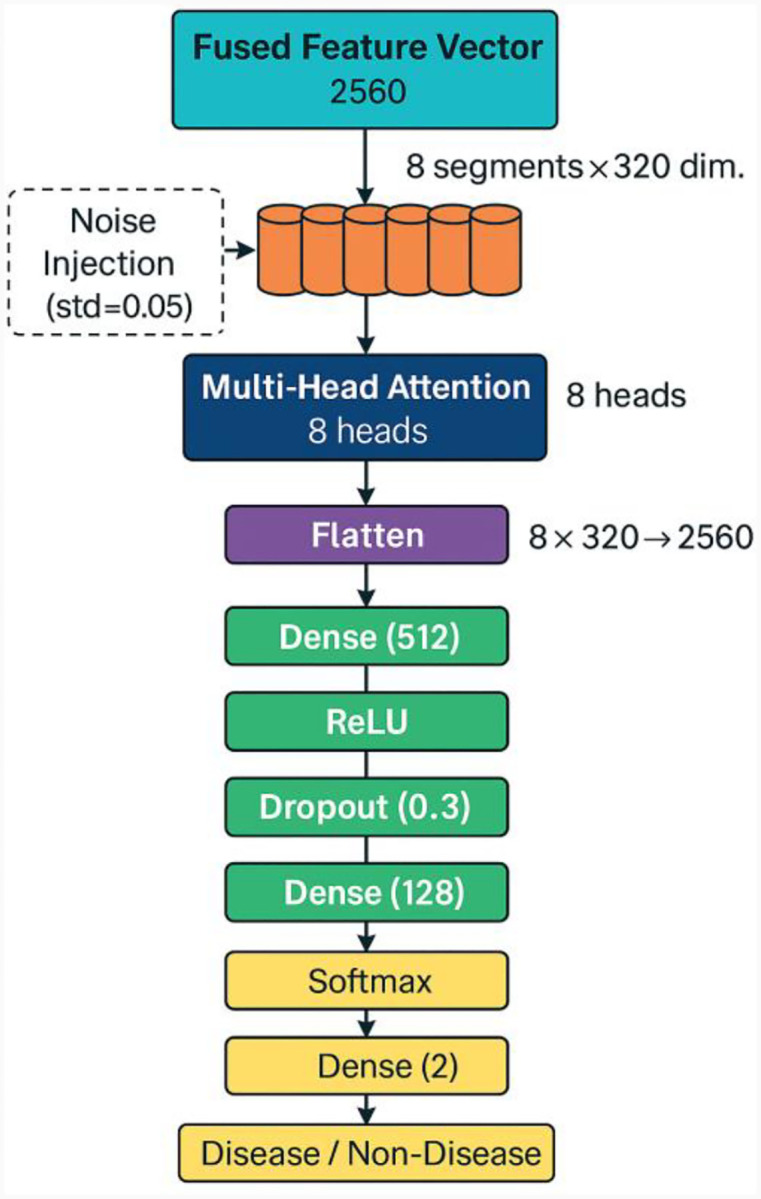
Architecture of the proposed attention-based multilayer perceptron (Attn-MLP) classifier. The fused feature vector (dimension 2560) is segmented into eight tokens of size 320 and passed through a multi-head self-attention mechanism with 8 heads. A lightweight noise injection layer (std = 0.05) is applied during training. The attention output is flattened and processed by two dense layers with ReLU activation and dropout regularization. A SoftMax classifier produces the final binary output indicating the presence or absence of disease.

Two fully connected layers with dropout regularization and ReLU activations were applied to the flattened attended representation. To increase robustness, Gaussian noise was introduced during training by a lightweight feature augmentation module. The last dense layer used SoftMax activation for probability estimation after projecting the hidden representation into two units that represented the binary classes. The architecture of the proposed classifier is illustrated in Figure 4. It successfully distinguishes between diseased and non-diseased cases by processing the fused features through a multi-head attention mechanism and a series of dense layers.

The AdamW optimizer was employed to optimize the model for 500 epochs, with a learning rate of 10e−4 and learning rate scheduling via cosine annealing. The final evaluation was conducted using the model that demonstrated the highest level of performance for each fold, as designated by the validation accuracy.

## 4 Results

Skeletal muscle pathology is distinguished by structural disruptions in sarcomeres, increased central nuclei, and changes in myofiber cross-sectional area. In order to classify amyotrophic lateral sclerosis (ALS), diabetes, and healthy controls, we examined the changes in myofiber size using Wheat Germ Agglutinin (WGA) stained histopathological images of various skeletal muscles (quadriceps, gastrocnemius, tibialis anterior, extensor digitorum longus, and soleus).

To automate this classification process, we developed the AFTG-Net model, which can learn discriminative features from patterns found in histopathology. In order to assess the effectiveness of our suggested AFTG-Net model for skeletal muscle pathology classification, we carried out thorough comparisons with a number of baseline and cutting-edge techniques, such as both conventional feature-based and deep learning models. WGA-stained histopathological images of various muscle types were used in the classification task in order to differentiate between ALS, diabetic, and healthy skeletal muscle tissue.

For all traditional approaches using XGBoost, we conducted hyperparameter tuning to ensure optimal performance. Parameters such as the number of estimators, learning rate, maximum depth, and regularization coefficients were systematically adjusted using cross-validation. The best-performing configurations, identified through validation performance, are summarized in Table 1.

We fine-tuned a series of convolutional neural network (CNN) architectures, including ResNet50, EfficientNetB0, EfficientNetB1, EfficientNetB2, and InceptionV3, all initialized with pre-trained weights from the ImageNet dataset. For each architecture, the top five layers were removed, and the remaining layers were either frozen or selectively fine-tuned to allow adaptation to the domain-specific task while preserving general visual features learned from large-scale natural image data. Before processing, input images were resized to 224 × 224 pixels. Each CNN’s final pooling layer was used to extract feature representations, which were then sent through a specially designed classification head that included a 64-unit fully connected layer, a ReLU activation, and a regularization dropout layer. The Adam optimizer was used to train the models with a batch size of 64, a learning rate of 1e-4, and a maximum of 100 epochs. The best-performing model weights were restored by applying early stopping with a 10-epoch patience to avoid overfitting. If the validation loss did not improve over five consecutive epochs, a learning rate scheduler was used to lower the learning rate by a factor of 0.5, with a minimum learning rate threshold of 1e-6.

**Figure 5.**
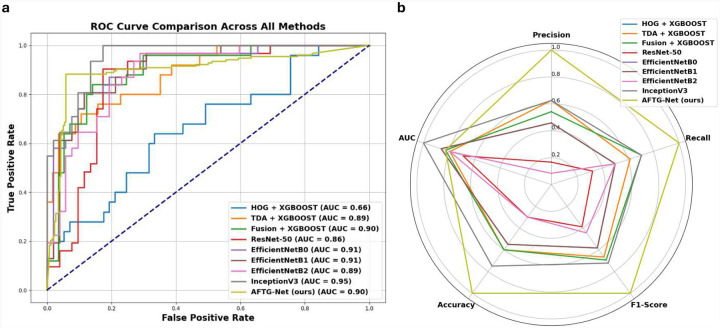
**(a)** ROC curve comparison of the proposed AFTG-Net against conventional machine learning methods (e.g., HOG + XGBOOST, TDA + XGBOOST) and deep learning models (ResNet-50, EfficientNet variants, and InceptionV3). **(b)** Radar plot comparing normalized performance metrics (Precision, Recall, F1-Score, Accuracy, and AUC) for all methods. AFTG-Net demonstrates superior and consistent performance across all evaluation metrics.

AFTG-Net consistently outperformed all competing methods across critical evaluation metrics, such as accuracy, precision, recall, and F1-score, as Table 2 and Figure 5 demonstrate. HOG + XGBOOST had the lowest overall performance among the conventional classifiers, with an F1-score of 0.56 and an AUC of 0.66. A significant improvement was obtained by substituting topological features (TDA + XGBOOST) for low-level texture features (F1-score: 0.81, AUC: 0.89). Combining both feature types (Fusion + XGBOOST) resulted in further improvement, with an F1-score of 0.80 and an AUC of 0.90. Nevertheless, the suggested AFTG-Net, which uses an attention mechanism to adaptively weight topological and gradient features, performed better than all conventional models, obtaining the highest accuracy (0.92 ± 0.061) and F1-score (0.92).

Deep learning models, including ResNet-50 and EfficientNet variants, demonstrated moderate to high AUCs (0.86–0.91); however, they suffered from by lower recall or F1-scores, which were typically in the 0.70–0.77 range. For instance, ResNet-50 achieved an F1-score of 0.70, while EfficientNetB0 and B1 achieved 0.77. On the other hand, InceptionV3 demonstrated superior performance, attaining the highest AUC (0.95) and an F1-score of 0.82 among all models, suggesting a robust ranking capacity. Nevertheless, it continued to lag behind AFTG-Net in terms of precision, recall, and cross-validated accuracy, indicating that AFTG-Net provides more consistent and threshold-robust classification performance than InceptionV3, despite being a competitive baseline.

In general, these results verify the advantages of gradually enhancing feature representations: beginning with low-level gradients, incorporating topological summaries, combining both through feature fusion, and ultimately learning adaptive weights through attention. The suitability of AFTG-Net for the robust classification of skeletal muscle pathology is underscored by its consistent performance across folds and evaluation metrics.

As illustrated in Figure 5a, AFTG-Net achieves competitive AUC scores compared to state-of-the-art models, while maintaining more stable and balanced classification performance across folds. Furthermore, the radar plot in Figure 5b highlights AFTG-Net’s consistent superiority across all core evaluation metrics, including precision, recall, and F1-score, underscoring its robustness for pathological image classification.

**Figure 6.**
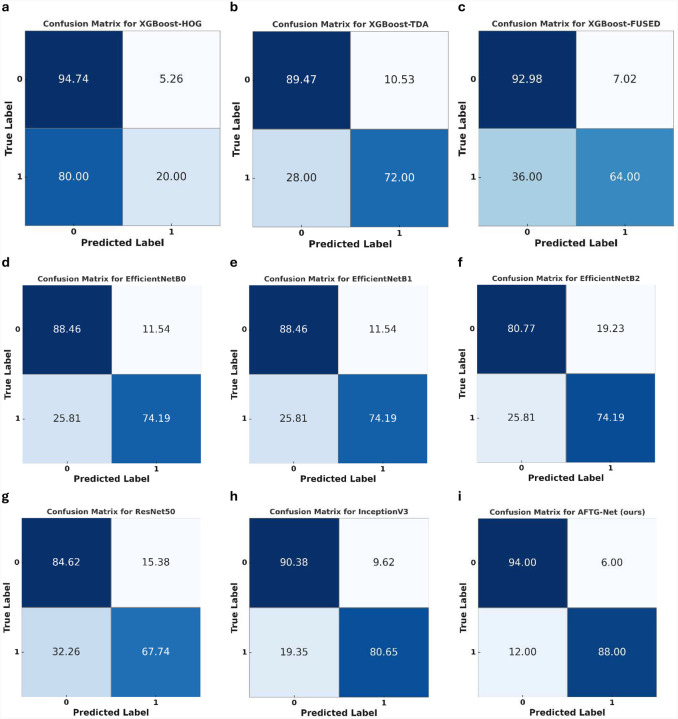
confusion matrices (percentages) for all evaluated models. **(a)** HOG + XGBOOST, **(b)** TDA + XGBOOST, **(c)** Fusion + XGBOOST, **(d)** EfficientNetB0, **(e)** EfficientNetB1, **(f)** EfficientNetB2, **(g)** ResNet-50, **(h)** InceptionV3, and **(i)** AFTG-Net (ours). Each matrix shows the classification performance across two classes, normalized by true label count. AFTG-Net demonstrates the highest per-class accuracy with minimal misclassification, particularly for class 1, compared to both traditional feature-based models and deep learning baselines.

The proposed AFTG-Net achieves the most balanced and consistent classification across both classes, as illustrated in Figure 6, with 94% of class 0 and 88.00% of class 1 samples correctly classified. This is significantly higher than competing methods, particularly in class 1, where numerous models demonstrate diminished sensitivity. For instance, XGBoost-HOG misclassified 80% of class 1 samples, and even strong models like ResNet-50 and EfficientNetB2 showed false negative rates above 25% for class 1. Compared to AFTG-Net, InceptionV3 demonstrated superior symmetric performance (90.38% for class 0 and 80.65% for class 1), but it still fell short in terms of per-class accuracy and overall robustness. The benefits of attention-guided fusion of topological and gradient features in managing inter-class variability and ensuring classification consistency across pathological classes are underscored by these findings.

## 5 Discussion and Conclusions

WGA staining of skeletal muscle images reveals a number of pathological features, including heterogeneous fiber size, an increase in myofibers with central nuclei, and fiber size variation across all muscle groups. These changes indicate the disease’s progression and are accompanied by the activation of inflammatory pathways and fibrotic remodeling. Muscle biopsy is the gold standard for numerous skeletal muscle diseases, including inflammatory, vascular, and congenital myopathies [57]. However, it is unfortunately susceptible to human error, as evidenced by interlaboratory deviations in measurement and inter-pathologist disagreements in certain instances [58]. In general, the calculation of fiber size necessitates manual pixel annotation, which are labor-intensive, time-consuming, and susceptible to user variability. ImageJ-based tools, which are commonly used to analyze skeletal muscle pathology, rely heavily on manual processes such as cell counting, segmentation, and thresholding. These methods require a lot of labor and are prone to inter- and intra-user variability, which may affect diagnostic accuracy.

On the other hand, AFTG-Net automates these tasks, thereby minimizing subjectivity and reducing the time required for analysis. Our method eliminates manual annotation that is capable of autonomously identifying and classifying these pathological changes. The significance of this automation is especially evident in neuromuscular and metabolic disorders, such as diabetes and ALS, where the diagnostic hallmarks are subtle morphological changes in muscle fibers, such as central nucleation or fiber atrophy. Our findings are consistent with previous research that has pointed out the limitations of manual histopathology and the increasing importance of machine learning in biomedical imaging. In addition, errors that occur during the preparation process, such as incomplete staining and sample defects, can result in errors [58]. Automated and machine learning models are required to assess the histology of human skeletal muscle, as these issues also arise during the classification of human skeletal muscle [59].

Our experiments show that AFTG-Net consistently outperformed traditional machine learning models and standard deep learning architecture on all key evaluation metrics. Traditional classifiers based solely on HOG underperformed, most likely due to the descriptor’s limited ability to capture global structural changes present in pathological muscle. The performance was enhanced by the integration of global topological information into TDA, and additional improvements were made by combining both HOG and TDA features using our proposed cross-weighted fusion strategy. AFTG-Net’s attention mechanism enabled the adaptive weighting of these features, which led to more discriminative representations that were in good alignment with the underlying muscle pathology. This is particularly noteworthy.

Among deep learning models, InceptionV3 had the highest AUC (0.95) but a lower F1-score and recall than AFTG-Net. This suggests that, while pre-trained CNNs can recognize certain visual features, they may struggle to generalize to fine-grained structural distinctions in muscle histology, especially when trained on natural image features. In contrast, our model’s ability to incorporate domain-relevant features directly, coupled with attention-guided fusion, enabled more consistent classification across disease classes. This was especially evident in the confusion matrices, where AFTG-Net achieved the most balanced accuracy across both classes and exhibited the lowest false negative rate for disease detection.

The robustness and generalization performance of AFTG-Net were further supported by its low standard deviation in accuracy across 20 folds, suggesting its potential for real-world deployment in diagnostic pipelines. The success of our fusion approach also highlights the importance of domain-aware feature engineering in medical image analysis, where handcrafted features may still offer value, especially when combined with learning-based models.

The implications of AFTG-Net extend beyond research and into clinical practice. By reducing the time and specialized expertise required for histopathological analysis, AFTG-Net enhances scalability, enabling large-scale screening of patient populations and facilitating longitudinal studies of disease progression. This is especially important for ALS, where early diagnosis remains challenging, and for diabetes, where skeletal muscle pathology is frequently overlooked. In addition, the model’s capacity to mitigate inter-observer variability has the potential to standardize diagnostic criteria across institutions, a challenge that has persisted for an extended period in the study of pathology. AFTG-Net’s rapidity and efficiency may contribute to the reduction of the scarcity of pathologists by optimizing diagnostic workflows [60]. In low-resource settings, where the disease burden is high but access to pathologists and laboratory services is limited, automated tools such as AFTG-Net can expand access to histopathological analysis [60]. Lastly, the automation offered by AFTG-Net in research environments can expedite histological classification, providing a faster alternative to manual tools like ImageJ.

However, this study is subject to a few limitations. First, the dataset is restricted in size and diversity in comparison to real-world clinical data, despite the fact that it contains multiple anatomical muscle regions and two disease models. Secondly, the cross-weighted fusion strategy is effective; however, it operates in a static (non-learnable) manner. Future research may investigate end-to-end trainable fusion modules that jointly optimize feature integration during model training. Third, while our current classification system is binary (healthy versus diseased), the clinical utility could be improved by further stratification into specific disease types and severity grades.

## Figures and Tables

**Table 1.** Optimal XGBoost hyperparameters selected for each feature-based method using cross-validation. Parameters include the number of estimators, maximum tree depth, learning rate, and subsampling ratio. Each configuration was chosen to maximize validation performance for its corresponding feature set.

Method	n_estimators	max_depth	learning_rate	subsample
HOG + XGBOOST	300	7	0.01	0.6
TDA+ XGBOOST	200	6	0.05	0.8
FUSED +XGBOOST	200	5	0.05	0.8

**Table 2.** Comparison of classification performance across traditional feature-based models and deep learning architectures on WGA-stained skeletal muscle images. Metrics reported include precision, recall, F1-score, accuracy (with standard deviation where applicable), and AUC. The proposed fusion with attention-based model outperformed all baselines in all evaluation criteria using 20-fold cross-validation.

Method	Train/Test	Precision	Recall	F1-Score	Accuracy	AUC
HOG + XGBOOST	20-fold	0.68	0.57	0.56	0.72 (± 0.049)	0.66
TDA+ XGBOOST	20-fold	0.81	0.81	0.81	0.84 (± 0.041)	0.89
Fusion + XGBOOST	20-fold	0.83	0.78	0.80	0.84 (±0.039)	0.90
EfficientNetB0	80/20	0.79	0.74	0.77	0.83	0.91
EfficientNetB1	80/20	0.79	0.74	0.77	0.83	0.91
EfficientNetB2	80/20	0.70	0.74	0.72	0.78	0.89
ResNet-50	80/20	0.72	0.68	0.70	0.78	0.86
InceptionV3	80/20	0.83	0.81	0.82	0.87	**0.95**
AFTG-Net (ours)	20-fold	**0.92**	**0.91**	**0.92**	**0.92** (±0.061)	0.90

## Data Availability

All datasets generated and analyzed during the previous study are available from the corresponding author on reasonable request.
